# A theoretical framework for linking hospitals longitudinally: demonstrated using German Hospital Quality Reports 2016–2020

**DOI:** 10.1186/s12874-024-02317-z

**Published:** 2024-09-19

**Authors:** Limei Ji, Max Geraedts, Werner de Cruppé

**Affiliations:** Institute for Health Services Research and Clinical Epidemiology, Philipps-Universität Marburg, Karl-von-Frisch-Strasse 4, 35043 Marburg, Germany

**Keywords:** Longitudinal data linkage, Linking hospital-level data, Hospital identity, Hospital variables, Similarity matrix, German hospital quality report, Minimum caseload requirements

## Abstract

**Background:**

In longitudinal health services research, hospital identification using an ID code, often supplemented with several additional variables, lacks clarity regarding representativeness and variable influence. This study presents an operational method for hospital identity delimitation and a novel longitudinal identification approach, demonstrated using a case study.

**Methods:**

The conceptualisation considers hospitals as evolving entities, identifying “similar enough” pairs across two time points using an automated similarity matrix. This method comprises key variable selection, similarity scoring, and tolerance threshold definition, tailored to data source characteristics and clinical relevance. This linking method is tested by applying the identification of minimum caseload requirements-related German hospitals, utilizing German Hospital Quality Reports (GHQR) 2016–2020.

**Results:**

The method achieved a success rate (min: 97.9% - max: 100%, mean: 99.9%) surpassing traditional hospital ID-code linkage (min: 91.5% - max: 98.8%, mean: 96.6%), with a remarkable 99% reduction in manual work through automation.

**Conclusions:**

This method, rooted in a comprehensive understanding of hospital identities, offers an operational, automated, and customisable process serving diverse clinical topics. This approach has the advantage of simultaneously considering multiple variables and systematically observing temporal changes in hospitals. It also enhances the precision and efficiency of longitudinal hospital identification in health services research.

## Background

Health services research evaluates the provision of health services by analysing the quality of health care to inform health policy decisions. A reliable evaluation of health service provision often extends over a long period of time and requires longitudinal studies. These studies are instrumental in examining changes or trends, and assessing the impact of exposures or interventions over time on research subjects, such as patients, households, or hospitals. [1] Studies in health services research often use administrative data as a secondary data source for analysis. These datasets usually cover annual periods, requiring a reliable linking approach to create multi-year datasets for longitudinal analysis.

Identifying research subjects consistently over time, referred to as linking data longitudinally, is imperative in such studies, especially in patient- or record-level medical research. [2–9] Although personal ID codes are effective for patient identification, they are often unavailable owing to data protection concerns. To overcome this limitation, personal variables like date of birth, sex, and postcode are commonly used for patient identification. By contrast, hospital ID codes are frequently available for hospital-level data and can be directly used as hospital identifiers (see also Table 1). However, these codes may not always reflect the hospital’s identity, which becomes particularly complex during changes in the hospital’s name, ownership, organisational type, address, medical departments, or ID code. Therefore, additional hospital variables are typically necessary for accurate identification. [10] There are six basic forms of hospital continuity: constant, with change in variables, new opening, closure, division, and merging. Using different combinations of variables to identify hospitals may result in diverse forms of continuity, even for the same situation. Defining a hospital’s identity is a critical consideration in hospital-level research, and patient-level linking approaches can offer insights into hospital-level identification.

Patient-level identification methods are categorised as deterministic, probabilistic, and advanced algorithms. Deterministic methods include rule-based processes such as decision trees, which are characterised by low workload and high requirement of logic clearance between the variables. Probabilistic methods employ score-based processes, such as similarity matrices, which simultaneously observe all integrated variables. Consequently, they entail a higher workload, but the logic between the variables is simplified using quantified probabilities. Advanced algorithms, such as data training/machine learning, require high effort and resources during the training process. This approach enables the logic between the variables to be identified through data processing, rather than being defined by researchers. [4, 8] In patient-level identification, the choice of method is determined by the workload and the expected level of accuracy. At the hospital-level, however, the choice of method depends on the understanding of the hospital’s identity, i.e., employing an algorithm to ascertain the logic behind the identity decision-making process.

Published hospital-level longitudinal studies [10–15] exhibit variations in the multiple dimensions of understanding and addressing the complexity of longitudinal hospital identity and identification (see Table 1). All the studies conceptualised the hospital identity as an administrative unit defined by a specific combination of identification code, location, and ownership [10, 13–15] (see “Referred variables for hospital identification” and “Longitudinal hospital identification approach” in Table 1). In studies where the hospitals were not necessarily linked individually, they were linked clustered, and the cluster variables were used for linking [11]. In studies where the hospitals were linked individually, the linking process was reported as a preparative step [12–14] or as the main work of the research [10, 15] (see “Use of hospital identity” in Table 1). As a preparative step, the linking principles were either simplified, for example using only the hospital ID [12], or reported as using hospital information in general [13, 14]. Studies that reported the linking process as the main work described the complexity of the task and reported success rates. These studies often attributed these success rates to the data source quality or the multidimensional characteristics of hospital change, without delving into the specifics of the linking method, or its reusability and generalisability. [10, 15]

This study supplements existing studies by providing both analytical theory and an automatable operational procedure to establish longitudinal identification at the hospital level, thereby advancing health services research.

**Table 1:** Hospital-level longitudinal studies and delimitation of hospital identity

Researched period	Authors	Hospitals are clustered, or individually linked	Hospitals linked across or within dataset	Referred variables for hospital identification	Longitudinal hospital identification approach	Use of hospital identity
**The evolution of hospital systems: unfulfilled promises and self-fulfilling prophesies**						
1979–1985 [USA]	Shortell [11]	Clustered linked	Within dataset	Clustered according to hospitals’ ownership	Hospitals are clustered by ownership and the form of organisation at different time points.	Linking hospitals as a preparative step. Each cluster’s proportional change is analysed. Hospitals need not be linked individually.
**Minimum caseload requirements and in-hospital mortality: observational study using nationwide hospital discharge data from 2006 to 2013**						
2006–2013 [Germany]	Nimptsch et al. [12]	Individually linked	Within dataset	Institution identification code	Hospitals are linked longitudinally only by institutional identification codes, without observing other variables.	Linking hospitals as a preparative step. Hospitals are linked, and data are pooled for the statistics.
**Longitudinal analysis on the development of hospital quality management systems in the Netherlands**						
1995–2007 [The Netherlands]	Dückers et al. [13]	Individually linked	Within dataset	Unclear	Hospitals are manually identified in pairs across different years.	Linking hospitals as a preparative step. Hospitals are linked in the research period, and statistically analysed using multi-level analysis.
**Longitudinal analysis of high-technology medical services and hospital financial performance**						
2005–2010 [USA]	Zengul et al. [14]	Individually linked	Within and across datasets	Hospital information	Hospitals are identified across datasets in order to combine variables from different data sources.	Linking hospitals as a preparative step. The characteristics of the longitudinally linked hospitals are included in the statistics. Variations between hospitals, and within individual hospitals over time are analysed.
**Problems with using hospital quality reports as a secondary data source for health services research in Germany**						
2006–2012 [Germany]	Kraska et al. [15]	Individually linked	Within dataset	1) Institution identification code 2) Address	Hospitals are longitudinally linked if the hospitals in the research period have the same identification code, or the same address but a changed identification code because of entering or leaving a hospital association.	Linking hospitals as the main work. The hospitals are observed individually with longitudinally linked datasets.
**Creating longitudinal hospital-level datasets**						
1982–2001 [USA]	Remy et al. [10]	Individually linked	Within and across datasets	Multiple variables, e.g.: hospital identifiers, location, ownership, organisational type, structural capacity	Linking hospitals longitudinally by comparing hospital variables and searching for further hospital information if needed	Linking hospitals as the main work. Hospitals are studied for their similarities for the longitudinal continuity, as well as changes in their status, such as opening, closure, relocation, consolidation, and other administrative changes.
**A theoretical framework for linking hospitals longitudinally: Demonstrated using German Hospital Quality Reports 2016–2020 (current research)**						
2016–2020 [Germany]	Ji et al. (current research)	Individually linked	Within dataset	1) Institution identification code 2) Location	Hospital identification codes and hospital locations are used to identify hospitals longitudinally.	Linking hospitals as the main work. Hospitals are considered as service providers for a certain regional area, to observe the changes in hospital distribution due to certain policies.

## Methods

**Fig. 1:**
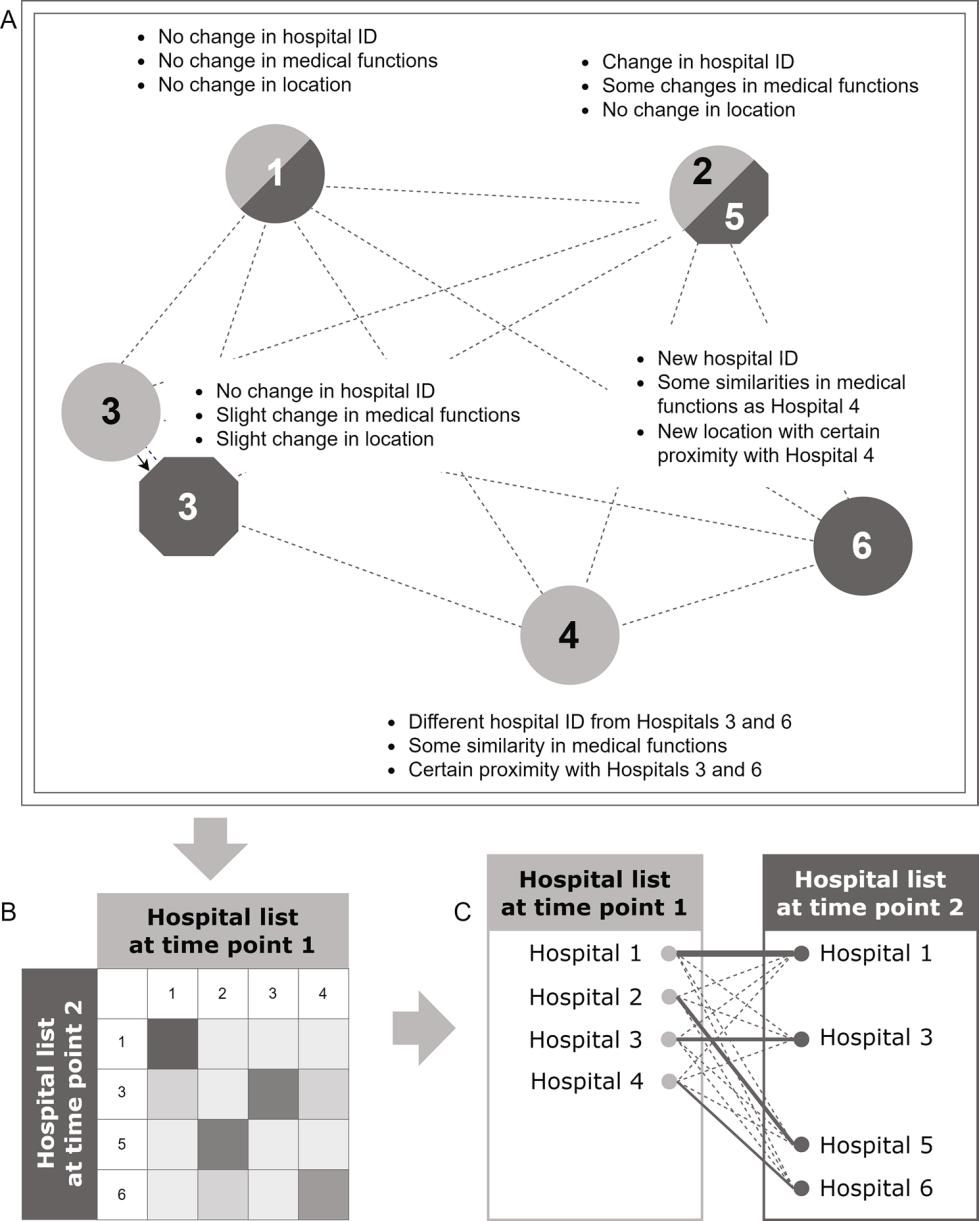
Schematic illustration of longitudinal hospital linking process with pseudo-map **(A)**, similarity matrix **(B)**, and linking decisions **(C)**. **(A)** Light grey circle: hospitals at the previous time point, e.g. 2016; dark grey circle/semicircle: hospitals at the next time point, e.g. 2017, without any change in administrative or functional characteristics; dark grey octagon/half octagon: hospitals at the next time point, with some changes in administrative or functional characteristics. **(B)** the greyscale indicates the degree of similarity between the hospitals at the two time points, with darker shades representing higher similarity and lighter shades representing lower similarity. **(C)** The thickness of the lines in the matrix corresponds to the degree of similarity between the hospitals. The solid lines represent the final linkages, while the dashed lines represent linkages that were not included in the final analysis due to low-ranking similarity

**Fig. 2:**
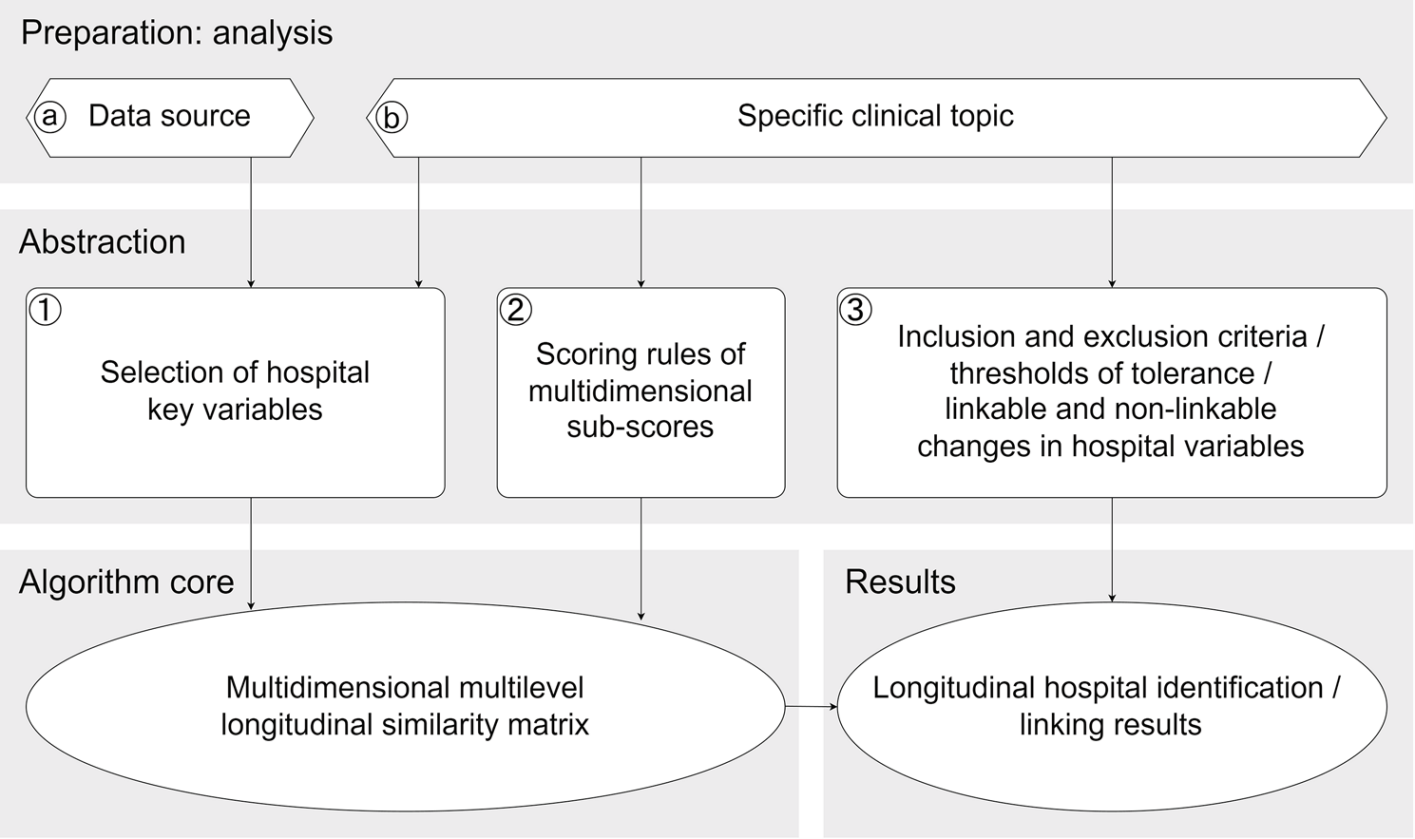
Work modules pertaining to analytical preparation, abstraction process, algorithm core and results of longitudinal identification of hospitals

Identifying or linking hospitals longitudinally involves discerning identical or similar hospitals at different time points. Various dimensions contribute to hospital changes and similarities, making the identification task complex (Fig. 1A). These dimensions encompass factors such as the hospital’s name, ID code, address, ownership, organisational type, number of beds, medical functions based on the medical department, and operations performed. These dimensions, typically available as hospital variables across diverse data sources, play a crucial role in the identification process. Based on this observation, a similarity matrix is used as the primary organisational tool for hospital comparison in this study. This matrix succinctly captured the similarity calculations for each hospital pair across selected variables (see Fig. 1B). Linkable hospitals were then identified through the application of a similarity matrix (see Fig. 1C). The similarity matrix represents the algorithm core of this method, which is underpinned by two preparative analyses (ⓐ, ⓑ) and three abstraction modules (①-③) (see Fig. 2).

To prepare the linking process, both ⓐ the data source characteristics and ⓑ the specific clinical topic must be analysed in order to delimit the hospital identity. This analytical preparation serves as the foundation for the decision-making process, which includes three distinct work modules for information abstraction. ① Selection of key hospital variables from existing data, guided by the preparative work of ⓐ and ⓑ; ② definition of scoring rules aligned with the requirements of the specific clinical topic using the selected variables; and ③ determination of similarity thresholds to translate the degree of similarity into decisions regarding hospital longitudinal identification. These thresholds, reflecting the identification tolerance and substitutability of hospitals over changes in the variables adjusted for related clinical topics, were then applied to establish longitudinal linkages (see Fig. 2).

The operation of our method is demonstrated in detail through a case study, which links hospitals longitudinally using German hospital quality reports (GHQR) [16] 2016 to 2020 as the data source. The linking results serve the specific clinical topic, that is, the analysis of minimum caseload requirements (MCR)-related health-service distribution and change.

### Analysis of the specific clinical topic

The continuity of hospital identity can be represented by different combinations of hospital variables, depending on the research topic. In this case study, we delimit hospital identity according to the following analysis.

Minimum caseload requirements (MCR) are legally mandated regulations that aim to regulate hospitals on medical/surgical interventions whose outcome quality is associated with the caseload. [17–21] MCR specify the required minimum annual caseload of these interventions in a hospital and allow compliant hospitals to perform them in the following year. In 2004 and 2006, six MCR interventions were introduced in Germany, including complex oesophageal and pancreatic interventions, stem cell transplantation, total knee replacement, liver transplantation, and kidney transplantation. This study analyses four of the MCR interventions: complex oesophageal and pancreatic interventions, stem cell transplantation, and total knee replacement. The MCR for liver and kidney transplantation are excluded due to their high concentration in approximately 20 and 40 hospitals respectively, constantly complying with the MCR. [19] The linking work for the four studied MCR interventions serves to evaluate the MCR compliance of the hospitals between 2016 and 2020. Approximately 1200 hospital sites that underwent at least one of the four studied MCR interventions, in at least one year during this period, were the subjects of this case study. These hospitals are mainly medium- to large-sized and are relatively longitudinally stable.

In this case study, hospitals were delimited as MCR service providers in a certain regional area. The main concern of hospitals’ continuity in this exemplary clinical topic under study is the provision of hospitals’ function and regional coverage. Ultimately, in this case study, the nearest hospitals across two years providing comparable MCR services in the same city/town should be delimited as longitudinally identical hospitals.

### Data source analysis and hospital variables selection

The purpose of the data source analysis was to determine the most suitable set of hospital variables for measuring similarity. The selection of key variables was based on a priori knowledge of the related clinical topic and thorough observation of the dataset.

We characterised the hospital variables into three aspects: (1) distinction of data, using the frequency of distinct values of the variables; (2) value stability, using the frequency of consistent values at two time points; and (3) variables’ interrelationships, i.e. the degree of the correlation of the selected variables, referring to the redundant or further distinctive information when selecting an additional variable for linking. The degree of variables’ interrelationship is mainly based on knowledge of the health system and the related documentation system. (See Table 2)

To identify hospitals with high reliability and low effort and resources, variable selection should combine variables with high distinction, high value stability, and low inter-relationships. These three principles are supplemented by knowledge of the topic relevance and clinical importance of the variables.

In this case study, GHQR data from 2016 to 2020 were used as the main data source. The GHQR are self-reported data from hospitals. They have been collected and openly published by the Federal Joint Committee every other year since 2004 and annually since 2012. Hospitals provided the data in a structured form under legal requirements. The GHQR includes information such as the hospital’s ID, name, ownership type, address, medical department, number of beds, number of medical staff, and number of procedures performed, as well as external quality assessment data based on self-reported documentation. GHQR is the only official data source for hospital MCR compliance. Approximately 2300 GHQR reports are published in Germany annually. In 2020, this number increased to 2538 due to the hospital site-specific reporting obligation. [17] Some large hospitals that were previously reported as a single entity are now reported as multiple sites as requested. For the current case study, the data source analysis (step ⓐ in Fig. 2) is carried out using GHQR 2016 to 2020. The most significant results for the variables commonly used in similar studies are presented in Table 2.

Considering the abovementioned three selection principles of high distinction, high value stability, and low inter-relationships, two variables were selected: hospital identification code and hospital location (see Table 2). The hospital identification code comprises an institution identification code (IIC) and a site code (SC). The IIC is an officially assigned code by the social care system in Germany to all hospitals and other social care providing institutions, serving for remuneration from the statutory health insurance in the German healthcare system. The IIC defines a hospital as a remuneration entity which may include more than one site. A hospital with only one site is referred to as a single hospital here, while a hospital with more than one site as a hospital association. The SC distinguishes hospital sites within a hospital association, and together with the IIC, each hospital site can be coded. A hospital with changes in the IIC or SC code is indicative of a hospital’s change in other variables, to a certain extent. [22] Since GHQR 2020, a new and official SC code system was introduced by the Federal Joint Committee, whereas the former SC code was set by each hospital association. The new SC can indicate each hospital site even without the IIC. In addition, the hospitals’ medical departments could be useful for MCR-related identification. However, the four currently concerned MCR interventions are usually located within the hospital’s main site instead of satellite sites. Meanwhile, the main sites usually maintain the addresses of former hospitals when they develop or are divided into several sites. Based on these two interrelationships, the continuity of the MCR functions and related medical departments was highly associated with the continuity of the hospital location. Therefore, the variables for medical departments were omitted. Further variables, as listed in Table 2, were not used because of the high interrelationships between the selected variables and the low or medium distinction levels. During the study, we found that adding redundant key variables, which have a high degree of interrelationship with the selected variables, but without any further distinction, resulted in more effort in calculations but not necessarily an improvement in accuracy.

**Table 2:** Analysis and selection of hospital variables for linking longitudinal hospital-level data based on German Hospital Quality Reports (GHQR) data from 2016 to 2020

Hospital variables	(1) Distinction		(2) Value stability		(3) Interrelationship with other variables	
	Level	Number of distinct values in each year	Level	Number of hospitals with constant value of the variable	Level	rough estimation
**Hospital identification code**	**High**	**2275**^**a**^, **2297**^**b**^, **2300**^**c**^, **2299**^**d**^, **2538**^**e**^	**High**	**2198**^**f**^, **2211**^**g**^, **2230**^**h**^, **2203**^i*^	**Low**	**This variable is based on the health system and the related documentation system**,** and is hardly associated with other hospital variables.** **Certain parts of this variable are associated with the hospital’s main site.**
**Location**	**High**	**2200**^**a**^, **2222**^**b**^, **2221**^**c**^, **2210**^**d**^, **2384**^**e**^	**High**	**2224**^**f**^, **2237**^**g**^, **2250**^**h**^, **2192**^**i**^	**Low**	**The geo-coordinates are hardly associated with other hospital variables.** **Some indicators based on this variable are associated with city size and similar aspects.**
Medical department (MD)	Medium	MD-code combination: 1534^a^, 1540^b^, 1519^c^, 1507^d^, 1541^e^ number of MD: 50^a^, 48^b^, 49^c^, 48^d^, 50^e^	Medium	MD-code combination: 1606^f^, 1689^g^, 1651^h^, 1578^i^ number of MD: 1891^f^, 1912^g^, 1907^h^, 1793^i^	High	Some MDs are associated with the hospital’s main site; some others are rather associated with the satellite sites. The number of MDs associated with aspects such as the hospital’s main site, hospital size, and hospital ownership.
Hospital ownership	Medium	owner: 1480^a^, 1479^b^, 1471^c^, 1457^d^, 1458^e^ type of ownership: 3^a, b,c, d,e^	High	owner: 2032^f^, 2058^g^, 2101^h^, 2041^i^ type of ownership: 2223^f^, 2233^g^, 2237^h^, 2215^i^	High	This variable is associated with aspects such as hospital size and certain MDs.
Hospital size	Low	number of beds: 630^a^, 640^b^, 637^c^, 640^d^, 635^e^	Medium	number of beds: 1726^f^, 1666^g^, 1646^h^, 1653^i^	High	This variable associates with aspects such as the number of MDs and city size.
Academic / teaching hospital	Low	yes or no: 2^a, b,c, d,e^ teaching institutes: 386^a^, 393^b^, 405^c^, 405^d^, 430^e^	High	yes or no: 2304^f^, 2323^g^, 2321^h^, 2572^i^ teaching institutes: 2191^f^, 2192^g^, 2192^h^, 2310^i^	High	This variable associates with aspects such as hospital size.

For variable’s distinction: based on ^a^: GHQR 2016; ^b^: GHQR 2017; ^c^: GHQR 2018; ^d^: GHQR 2019; ^e^: GHQR 2020

For variable’s value stability: based on comparison between GHQR ^f^: 2016 and 2017; ^g^: 2017 and 2018; ^h^: 2018 and 2019; ^i^: 2019 and 2020

^*^: The value stability of hospital identification code: for 2016 and 2017, 2017 and 2018, 2018 and 2019, IIC-SC are compared; for GHQR 2019 and 2020, IIC are compared.

### Hospitals’ longitudinal similarity matrix: scoring rules

This procedure is performed stepwise for hospitals in each of the two following years (2016–2017, 2017–2018, 2018–2019 and 2019–2020), which are referred to as “year one” and “year two” in this section. Finally, the results were assembled for the years 2016–2020. The two selected key variables (hospital identification code and hospital location) were used to assign a score to all year one and year two hospital combinations in a similarity matrix. Each combination implies a possible linkage. A linkage with a higher score indicates higher similarity on the key variables, and thus a larger possibility to be the “correct” linkage (see Fig. 1). One or no linkage was then chosen as valid for each year one and year two hospital based on the scores.

The linking score is broken down into two sub-scores, each corresponding to one of the selected key variables: the IIC-SC score describes the similarity in IIC and SC, while the hospital location score describes the similarity in location. Both sub-scores ranged from 0 to 4.

*The IIC-SC score* was calculated by comparing *hospital administrative identification code*. In GHQR, this identification code consists of IIC and SC. Hospital *IIC-SC matrices* are used for scoring the linkages. The linkage is scored as two if the hospitals in years one and two have the same IIC or scored as four for the same IIC and SC combination. A different IIC obtained a sub-score of zero (see Table 3).

The comparison of *hospital locations* in years one and two was expressed as the distance of relocation. Instead of the absolute distance value, the *hospital location score* was assigned based on the ranking of the relocation distance. The calculation was based on *distance matrices*. The distances from year one hospitals to the nearest year two hospitals (forward linkages) and in the reverse direction (backward linkages) were calculated separately (Table 3) using the following steps. (1) Forward linkage: For each hospital A in year one, calculate its distance D to each hospital in year two. Find hospital B from year two such that the distance D_AB_ is the shortest among those between A and each hospital in year two. Linkage AB obtains a score of two if D_AB_ is zero; otherwise, it obtains a score of one; if multiple linkages have the same minimal distance, they are scored in the same way. All other linkages from A were scored as zero. (2) The backward linkage works in the same way as in years one and two’s swapped positions. (3) Each linkage between any hospital in year one and any hospital in year two had two scores from forward and backward linking. The sum of these is the sub-score for hospital locations. The possible scores were four, two, one, and zero. Hospital location coordinates were converted from the hospital addresses, primarily from GHQR, using the Google API geocoding function. In case of inaccurate address information, supplements are obtained from the “positive list” [23] 2016–2019 (list with all hospital sites from which GHQR are expected) from the Federal Joint Committee and the hospital site directory from the Institute for the Hospital Remuneration System (in German: Institut für das Entgeltsystem im Krankenhaus, InEK) [24] since 2019.

Inaccurate addresses were identified using the similarity matrix. If the addresses of the most similar hospitals differed, each address was individually verified by comparing the address provided in the other years’ GHQR, in the “positive list”, and on their official websites. This process determined whether the change in address was due to relocation or an inaccurate address in the GHQR of a certain year. Once the inaccurate addresses had been identified and corrected, the similarity matrix was regenerated. However, in the case of addresses that have been consistently mis-documented, this approach will not be effective.

**Table 3:** Longitudinal similarities: scoring rules for longitudinal linkages

Types of similarity / types of linkages	Sub-score
**IIC-SC score**	
Identical IIC-SC	4
Identical IIC, but different SC	2
Different IIC	0
**Hospital location score**	
Identical hospital address (relocation = 0)	4
Mutually the nearest hospitals (relocation > 0)	2
Only forward linkable, not backward linkable (relocation > 0)	1
Only backward linkable, not forward linkable (relocation > 0)	1
Neither of them is the nearest hospital to each other	0

### Thresholds of tolerance and linking decisions

Tolerance thresholds define the acceptable range of change in hospital variables. These thresholds vary depending on the specific clinical topic under consideration. In practice, the thresholds are set analytically and tested using several cases to assess the effect on linking results. In this case study, the maximum possible total score for linking is 8, indicating no change in the two selected key variables, IIC-SC and location, and a high probability of the same hospital at two points in time. Among all linkages to the same hospital in one year, the one with the highest score was considered valid. If the highest score appears more than once, the relevant hospitals will be manually checked using reference sources, such as other hospital variables from the GHQR and information from online websites about newly opened hospitals and hospital closures, and only one is chosen as the final linkage. If the selected linkage crosses a city/town, it is disregarded. Ultimately, each year one and year two hospital receives one linkage or none.

### Types of hospital identification results

In general, registered hospitals in Germany function primarily as inpatient treatment settings. Their opening, closure, merging, division, and restructuring are regulated by the German Social Code, Book V (SGB V). However, for specific research purposes, this definition of continuity is not always valid. Focusing on different aspects of hospitals leads to divergent conclusions regarding their continuity. Consequently, the researchers need to clarify the continuity of hospitals in the context of their specific clinical topic.

In accordance with the logical possibilities, there are six types of hospital longitudinal continuity. These are numbered consecutively as follows: type 1: constant, type 2: with change in variables, type 3: new opening, type 4: closure, type 5: division, and type 6: merging. In this study, the hospital division (type 5) is reconstructed as a slightly changed (type 2) and a newly opened hospital (type 3); and the hospital merge (type 6) as a slightly changed (type 2) and a closed hospital (type 4). The operational definition in the current MCR-related case study is listed in Table 4.

**Table 4:** Six types of hospital longitudinal continuity

Types of hospital continuity	Operational definition in the current MCR-related case study
(1) Constant hospital	No change in hospital IIC, SC, and location
(2) Hospital with change in variables	Still the same hospital but with changes in hospital IIC, SC and location, and the changes are within the tolerance area
(3) Newly opened hospital	No linkable hospital within the tolerance area in the past year
(4) Hospital closed	No linkable hospital within the tolerance area in the subsequent year
(5) Hospital division	Multiple linkages to one hospital in the past year, reconstructed as the combination of type 2 and type 3 in the current case study reconstructed
(6) Merged hospital	Multiple linkages to one hospital in the subsequent year, as the combination of type 2 and type 4 in the current reconstructed case study

### Validation of linking results

To evaluate the correctness of this method, we manually examined the concerned MCR-related linkages using (1) the hospital’s name, ownership type, medical departments, and number of beds from the GHQR, (2) the continuity of the four considered annual MCR caseloads, and (3) additional information on hospital closures, relocations, and transformations from websites. These three aspects were not used in the linking process, but are now used to determine the correctness of the linkages. The principle of determination is: if most of the three aspects remain constant or the change does not affect the MCR service provision, the linkage is valid. If the service does not remain in the linked hospital, the linkage is not valid.

Once the manual validation of the linking results has been completed, the final linkages can be identified as the “correct linkages”. Thereafter, the results from the automated process in the current case study and from the simple IIC-SC approach, are compared with the “correct linkages” to determine the success rates of the two different approaches.

## Results

The frequencies of hospital linkages with different scores—that is, the frequency of changes in the hospital identification code and location—are presented in Table 5.

### Hospital continuity 2016–2020 in Germany

One to fourteen locations (0.1–1.2%) per year were not linked due to new hospital openings or closures between 2016 and 2020. In the linked MCR hospitals, 92.9–97.8% were hospital sites without changes in hospital IIC-SC codes and locations. This value is lower for 2019–2020 owing to the SC naming system change and the non-mandatory input of “old SC code”. The remaining linkages of MCR-related hospitals involve changes in either the hospital IIC-SC (20–74 cases annually, 1.7–6.2%, sum of lines 9 and 13 in Table 5, the same below), location (5–9 cases annually, 0.4–0.8%, sum of lines 10 to 12), or both (1–2 cases annually, 0.1–0.2%, sum of lines 14 to 18). Regarding the two parts of the IIC-SC code, the IIC change (lines 13, 17, and 18) occurred 8–13 times (0.7–1.1%) each year, while 12–67 cases (1.0–5.6%) annually involved only the SC change (sum of lines 9, 14, and 16).

### Manual verification of automated results

The values marked with footnotes 4–7 in Table 5 are corrections based on manual verification. In most cases, the four MCR interventions were moved together to the same successor hospital in case of a change or division. However, from 2016 to 2020, in two cases, the four interventions were split into different successor hospitals, resulting in different numbers of false matches (Table 6).

The frequency of multiple highest scores was 1–4 times per year between 2016 and 2019. However, because of the SC code system change in 2019–2020, using the “old SC code” input resulted in more frequent tie scores (24 times). Hospital linkages with tie scores are manually identified.

### Success rate

Table 6 compares the success rate of this method with that when only the IIC-SC code was used for identification. The current method had an average success rate of 99.9% (minimum: 97.9%, maximum: 100%) for different years for different MCR interventions. This is better and more reliable than using only the IIC-SC code, with an average success rate of 96.6% (minimum: 91.5%; maximum: 98.8%). Regarding the absolute number of linkages, our method prevented a total number of 294 linkages from being missing. Across all reports, up to 5–10 hospital sites per year had inaccurate address inputs. This includes the kind of inaccuracy that does not occur consistently across the five years discovered while handling exceptional values in the distance matrices. It cannot be ruled out that there may still be other inaccuracies in the addresses within the GHQR.

**Table 5:** Longitudinal linking results of hospitals: frequency of linkable and non-linkable hospitals, frequency of IIC-SC code change, and hospital relocation

		2016–2017			2017–2018			2018–2019			2019–2020	
	2016		2017	2017		2018	2018		2019	2019		2020`
**All hospitals with GHQR** ^h^												
**Number of GHQR hospital site reports**^a h^	**2275**		**2297**	**2297**		**2300**	**2300**		**2299**	**2299**		**2538**
Number of GHQR reports with errors in address input ^h^	5		10	10		7	7		9	9		9
Frequency of multiple highest overall score of linkage		2			1			4			24	
**Subset of MCR related hospitals in GHQR** ^h^												
Number of MCR-related hospital sites in respective years ^h^	1156		1146	1146		1135	1135		1117	1117		1109
**Number of MCR-related hospital sites in 2016–2020**^h^	**1226**		**1226**	**1226**		**1218**	**1218**		**1206**	**1206**		**1210**
**Hospitals without linkages**^b^ (types 3 and 4)^*^	**7 (0.6%)**		**7 (0.6%)**	**9 + 1–1 (0.7%)** ^**d**^		**1 (0.1%)**	**14 (1.1%)**		**2 (0.2%)**	**9 + 1 (0.8%)** ^**e**^		**13 + 1 (1.2%)** ^**e**^
**Longitudinally linkable hospital pairs [as 100% for linkages with different scores]**		**1219**			**1217 + 1–1** ^**d**^			**1204**			**1197-1** ^**e**^	
[HL4-IIC2-SC2]^c^ score of 8: identical location, identical IIC-SC (type 1)^*^		1185 (97.2%)			1181 (97.0%)			1178 (97.8%)			1111 (92.9%) ^f^	
[HL4-IIC2-SC0]^c^ score of 6: identical location, identical IIC, different SC (type 2)^*^		10 (0.8%)			20 − 1 (1.6%) ^d^			12 (1.0%)			65 (5.4%)	
[HL2-IIC2-SC2]^c^ score of 6: similar location (two-way nearest), identical IIC-SC (type 2)^*^		6 (0.5%)			7 (0.6%)			5 (0.4%)			3 (0.3%)	
[HL1-IIC2-SC2]^c^ score of 5: similar location (one-way nearest), identical IIC-SC (type 2)^*^		0 (0.0%)			1 (0.1%)			0 (0.0%)			5 (0.4%)	
[HL0-IIC2-SC2]^c^ score of 4: identical IIC-SC (type 2)^*^		3 (0.2%)			0 (0.0%)			0 (0.0%)			1 (0.1%) ^g^	
[HL4-IIC0-SC0]^c^ score of 4: identical location (type 2)^*^		13 (1.1%)			8 (0.7%)			8 (0.7%)			9 (0.8%)	
[HL2-IIC2-SC0]^c^ score of 4: similar location (two-way nearest), identical IIC, different SC (type 2)^*^		2 (0.2%)			0 (0.0%)			0 (0.0%)			1 (0.1%) ^g^	
[HL1-IIC2-SC0]^c^ score of 3: similar location (one-way nearest), identical IIC, different SC (type 2)^*^		0 (0.0%)			0 (0.0%)			0 (0.0%)			0 (0.0%) ^g^	
[HL0-IIC2-SC0]^c^ score of 2: identical IIC, different SC (type 2)^*^		0 (0.0%)			0 + 1 (0.1%) ^d^			0 (0.0%)			2 − 1 (0.1%) ^e, f^	
[HL2-IIC0-SC0]^c^ score of 2: similar location (two-way nearest) (type 2)^*^		0 (0.0%)			0 (0.0%)			1 (0.1%)			0 (0.0%)	
[HL1-IIC0-SC0]^c^ score of 1: similar location (one-way nearest) (type 2)^*^		0 (0.0%)			0 (0.0%)			0 (0.0%)			0 (0.0%)	

a: Total number of GHQR of hospital / hospital site reports: at least one part of “Parts A, B, C except C1” or “Part C1” of GHQR is available

b: Reasons for hospitals being non-linkable: hospital openings, closures, division, consolidation, restructuring into nursing homes, or missing reports

c: Sub-score-coding: e.g. HL4: hospital location sub-score = 4; IIC2-SC0: IIC-SC sub-score = 2 + 0 = 2

d: A manual correction for all four kinds of concerned MCR-interventions: a hospital was consolidated. The second site’s address was taken over (linkage score of 6), but the MCR-related function was retained in the first site (linkage score of 2). According to the continuity of concerned MCR-functions, the first site in 2016 and the newly consolidated hospital in 2017 are linked

e: One linkage was manually rejected for all concerned MCR-interventions: the old location was closed; a new location was opened in the same city / town but without relevant MCR-function being taken over

f: A hospital is divided into several sites. The linkage with a score of 8 is valid for complex oesophageal and pancreatic interventions and stem cell transplantation but invalid for total knee replacement. The location for knee replacement is at another site of the same association (linkage score of 2) after division

g: A hospital is divided into several sites. The linkage with a score of 4 (line no. 12) is valid for complex oesophageal and pancreatic interventions but not for stem cell transplantation and total knee replacement. After hospital division, the location for stem cell transplantation is at another site of the same association (linkage score of 3, line no. 15), and the location for knee replacement is at a third site of the association (linkage score of 4, line no. 14)

h: *Abbreviations* *GHQR* German Hospital Quality Reports; *MCR* minimum caseload requirements

*: See the types of longitudinal continuity in Table 4

**Table 6:** Success rate of longitudinal hospital identification

		Total linkage number	Current method		Identification based on only IIC-SC code		
			True matches	False matches	True matches	False matches	Missed matches
Oesophagus*	2016–2017	480	480 (100.0%)	0 (0.0%)	467 (97.3%)	0 (0.0%)	13 (2.7%)
	2017–2018	439	438 (99.8%)	1 (0.2%)	423 (96.4%)	1 (0.2%)	15 (3.4%)
	2018–2019	417	417 (100.0%)	0 (0.0%)	412 (98.8%)	0 (0.0%)	5 (1.2%)
	2019–2020	383	382 (99.7%)	1 (0.3%)	352 (91.9%)	0 (0.0%)	31 (8.1%)
Pancreas*	2016–2017	683	683 (100.0%)	0 (0.0%)	665 (97.4%)	0 (0.0%)	18 (2.6%)
	2017–2018	674	673 (99.9%)	1 (0.1%)	652 (96.7%)	1 (0.1%)	21 (3.1%)
	2018–2019	638	638 (100.0%)	0 (0.0%)	626 (98.1%)	0 (0.0%)	12 (1.9%)
	2019–2020	614	613 (99.8%)	1 (0.2%)	573 (93.3%)	0 (0.0%)	41 (6.7%)
Stem cells*	2016–2017	104	104 (100.0%)	0 (0.0%)	101 (97.1%)	0 (0.0%)	3 (2.9%)
	2017–2018	101	100 (99.0%)	1 (1.0%)	97 (96.0%)	1 (1.0%)	3 (3.0%)
	2018–2019	97	97 (100.0%)	0 (0.0%)	94 (96.9%)	0 (0.0%)	3 (3.1%)
	2019–2020	94	92 (97.9%)	2 (2.1%)	86 (91.5%)	1 (1.1%)	7 (7.4%)
Knee*	2016–2017	1063	1063 (100.0%)	0 (0.0%)	1043 (98.1%)	0 (0.0%)	20 (1.9%)
	2017–2018	1053	1052 (99.9%)	1 (0.1%)	1027 (97.5%)	1 (0.1%)	25 (2.4%)
	2018–2019	1026	1026 (100.0%)	0 (0.0%)	1010 (98.4%)	0 (0.0%)	16 (1.6%)
	2019–2020	1017	1014 (99.7%)	3 (0.3%)	954 (93.8%)	2 (0.2%)	61 (6.0%)
Statistics: true / false linkage number and success rate for different years and different clinical topics		8883	min: 92	min: 0	min: 86	min: 0	min: 3
			max: 1063	max: 3	max: 1043	max: 2	max: 61
			sum: 8872	sum: 11	sum: 8582	sum: 7	sum: 294
		as 100%	min: 97.9%	min: 0.0%	min: 91.5%	min: 0.0%	min: 1.2%
			mean: 99.9%	mean: 0.1%	mean: 96.6%	mean: 0.1%	mean: 3.3%
			max: 100.0%	max: 2.1%	max: 98.8%	max: 1.1%	max: 8.1%

*Abbreviated for complex oesophageal and pancreatic interventions, stem cell transplantation, and total knee replacement, respectively

## Discussion

Linking hospitals longitudinally is a typical preparatory task for longitudinal studies, particularly when using secondary data. Depending on the changes in the health care system, the linking work is often extensive, and carried out manually. In comparison with the previous studies [10, 13–15], this study added the awareness of the systematics of the linking work, the awareness of the workload due to the need to consider and monitor a multitude of variables simultaneously, and the awareness of the inherent complexity of the logical structure of these variables for decision-making in linking hospitals. Once these aspects are clearly defined, the subsequent work can be structured and automated. The focus then shifts from the tedious and laborious sorting and comparing of variable values as in previous studies [10, 13–15] to logic declaration. On this basis, we present a theoretical framework and an operational method for the automated longitudinal hospital linkage with customisable options.

To evaluate our method, we adopt the general linking principle of “using IIC-SC alone” as the comparison basis. We refrain from employing the concrete linking processes from other studies owing to the inherent differences in linking settings making their results incomparable with our MCR-focused research.

However, our case study has limitations, notably the exclusion of the hospitals’ medical departments as key variables. Calculations involving medical departments are complex owing to their non-mutually exclusive functions and intersecting roles, particularly in emergency cases. For example, knee replacements can be performed by the surgical, orthopaedic, or traumatology departments. In addition, medical departments are sometimes associated with other issues, like psychiatric day patient care in Germany, are often located in distinct satellite sites rather than the main hospital sites. In order to include the complex work of analysing and using medical department level information to determine hospital level continuity, the logic structure of medical department functions, the legit exceptions, the workload estimation, the possibility of automation and customisation, should all be evaluated. Nevertheless, while medical departments are vital for describing hospitals, their omission in this paper is acknowledged. Future studies should explore quantifying this variable for longitudinal hospital identification.

In 2018, the Institute for the Hospital Remuneration System initiated the longitudinal documentation of hospitals with newly assigned 9-digit site codes in Germany. The widespread adoption of this coding system by the main German hospital-related data sources in 2019 and 2020 underscores the significance of our proposed method, particularly in identifying hospitals during the transitional years before and after the site code system changes.

## Conclusions

This study establishes a theoretical framework for comprehending the hospitals’ longitudinal identities, acknowledging them as dynamic entities akin to the Theseus ship. The relevance, similarity, and substitutability of hospital variables contribute to the nuanced nature of the hospital’s longitudinal identities, which vary across clinical topics.

Building upon this conceptual foundation, we introduce an operational method for the automated longitudinal identification of hospitals with customisable options. The automated process substantially reduces the manual workload. The goal-oriented design ensures a low error rate in hospital linkages. Furthermore, this approach offers the advantage of simultaneously considering multiple variables and systematically observing their changes.

Each work module of this framework can be researched further in detail, from the conceptual framework to operationality using case studies, into a generalised operational quantification process. A software targeting this longitudinal linking process is foreseeable. The use of medical departments as one of the linking variables, along with workload, is to be researched.

## Data Availability

The GHQR data for this study are available from the Federal Joint Committee upon reasonable request. Processed data will be shared on request with the corresponding author with permission from the Federal Joint Committee.

## Abbreviations

MCR: Minimum caseload requirements
GHQR: German Hospital Quality Reports
IIC: Institution identification code
SC: Site code
